# Look and you will find—a literature review of new strains of *Leptospira* spp., 2000–2025

**DOI:** 10.1093/femsre/fuaf054

**Published:** 2025-11-06

**Authors:** Olena Pyskun, Martin H Richter

**Affiliations:** State Scientific Research Institute of Laboratory Diagnostics and Veterinary and Sanitary Expertise, Donetska Str., 30, 03151 Kyiv, Ukraine; Department Biological Safety, German Federal Institute for Risk Assessment, Max-Dohrn. Str. 8-10, 10589 Berlin, Germany

**Keywords:** leptospirosis, *Leptospira*, new strain, environmental, mammals, human, One Health

## Abstract

Leptospirosis is one of the most common zoonotic infections in the world and is considered a neglected disease. Development of molecular methods and approaches in gene typing significantly contributed to the discovery of novel *Leptospira* strains, which require detailed studying and systematization and are an important factor of managing the pathogen and the disease leptospirosis as a classic One Health problem. Characterization of *Leptospira* populations in water, soil, and other environmental objects will aid in the development and implementation of prevention and control approaches aimed at reducing the risks of infection, and will contribute to a deeper understanding of the bacteria’s ecology. This study aimed to briefly describe the phylogenic history of *Leptospira* spp., and to conduct a review and retrospective analysis of new strains discovered during the years 2000–2025 impacting the leptospires landscape significantly. The discovery of novel *Leptospira* strains has been an important development in the research of this pathogen, and has helped to better understand the potential risks associated with its presence. In this review, we analyzed and summarized literature on the detection of new *Leptospira* strains and their global distribution.

## Teaser

Review and retrospective analysis of 89 new *Leptospira* strains isolated during the years 2000–2025 and their impact on the leptospires landscape implicating an important trend of expanding sources for infection with the bacteria.

## Introduction

Leptospirosis is one of the most common zoonotic infections globally and is generally overlooked and under-reported as a neglected tropical disease (Allan et al. 2015, UNDRR 2025) but to this date is still not part of WHO’s official list of neglected tropical diseases (https://www.who.int/health-topics/neglected-tropical-diseases#tab=tab_1). This zoonosis is characterized by a wide range of hosts and exhibits a global distribution. In developing regions, leptospirosis outbreaks are expected to increase as the world’s population is projected to double to 8.5 billion by 2030 (Pappas et al. 2008). Urbanization, rapid suburban development, and inadequate sanitation systems create conditions conducive to the transmission of the pathogen, with rodents acting as the primary reservoirs.

Additionally, the spread of leptospirosis has been exacerbated by factors such as international trade, tourism, and migration due to war and poverty (Bandara et al. 2014). Climate change also plays a critical role, influencing the frequency and intensity of extreme weather events, including excessive rainfall, floods, and droughts, which facilitate the spread of leptospires through contaminated runoff into rivers and coastal waters (Suriya and Mudgal 2012).

In temperate regions, leptospirosis is endemic with seasonality, peaking in the summer through fall, where temperature is the limiting factor for *Leptospira* survival, and the number and frequency of disease outbreaks are related to outdoor activities and the amount of rainfall. In tropical countries, outbreaks of the disease are most often recorded during the rainy season, when temperature and humidity contribute to the reproduction and survival of pathogenic leptospires but the disease is present year-round (Munoz-Zanzi et al. 2020). Pathogenic leptospires thrive in environmental reservoirs such as ponds, rivers, sewage systems, and agricultural fields, where they can reproduce and persist (Cunha et al. 2022).

Rodents, particularly mice and rats are primary hosts. There leptospires typically do not cause disease, persist in the renal tubules, and are excreted with urine, contaminating the environment (Karpagam and Ganesh 2020). Humans and other warm-blooded animals, including farm livestock, are then mostly infected through direct contact with contaminated water or soil. Direct contact with contaminated urine is also possible. In rural areas, the disease presents a significant threat to farm animals, leading to both acute clinical manifestations and chronic infections that can result in severe systemic disease, economic losses, and reproductive issues such as abortions, stillbirths, and reduced milk production (Guglielmini et al. 2019).

For farmers, veterinarians, and workers in the meat processing industry, livestock is a key source of infection (Alinaitwe et al. 2024, 2025), while urban residents, hunters, forest workers, and herders are primarily exposed to wild animals and rodents (Mwachui et al. 2015). Subsistence farmers and rice workers with frequent exposure to water bodies and contaminated soils are particularly vulnerable (Soo et al. 2020). Furthermore, recreational activities involving contaminated water, poor sanitation in urban slums, and exposure to sewage increase the risk for travellers, water sports enthusiasts, people with occupations that cause frequent contact to the bacteria, and the general population.

According to statistics, there are more than 1 million cases of leptospirosis in the general population each year, which is a higher range than dengue fever or other tropical fevers with similar characteristics (Costa et al. 2015, Xu et al. 2017). In addition, underreporting of the disease is likely, as not all infections cause disease requiring treatment or even visits to a care physician. Another statistic states that every year, approximately 500 000 people show a severe course of the disease and the mortality rate ranges from 5% to 20% (Costa et al. 2015).

### Taxonomy, diagnostics, and classification of ***Leptospira*** spp.

The genus *Leptospira* (phylum Spirochetes) is genetically diverse and highly heterogeneous, with current classifications recognizing 78 species and several not validly published and unclassified species and over 300 serovars. This number is likely to continue to increase and current numbers can be found, for instance, in the National Institute of Health Taxonomy browser repository (Taxonomy Browser 2025).

Phylogenetic analyses have revealed that leptospires are divided into three main monophyletic clusters: “pathogens,” “intermediates,” and “saprophytes” (Plank and Dean 2000, Thibeaux et al. 2018, Arent et al. 2022).

The “pathogen” cluster includes life-threatening species such as *Leptospira* interrogans, the most prevalent pathogenic species worldwide, capable of infecting a wide range of hosts, including mammals, birds, and reptiles. This species owes its virulence to a range of virulence factors such as hyaluronidase, phospholipases, lipases, hemolysins, and endotoxins (Plank and Dean 2000).

The “intermediate” cluster, recently characterized in both humans and animals, includes strains with low virulence but significant genomic differences, as well as strains with unexplored virulence properties. These species exhibit unique genomic features that distinguish them from classic pathogens (Plank and Dean 2000, Thibeaux et al. 2018).

The “saprophyte” cluster consists of environmental species, which are typically cleared rapidly in animal models and are non-pathogenic to humans and other animals (Plank and Dean 2000, Thibeaux et al. 2018). Historically, it is believed that saprophytes evolved from free-living soil organisms to virulent species capable of infecting mammals, primarily through the expansion of specific protein families via gene duplication. Virulence evolved independently in pathogens and intermediates, as indicated by the distinct patterns of accessory genes and domains within these groups (Thibeaux et al. 2018).

Further taxonomic refinement, supported by infection studies and genome analyses, has led to the identification of two subgroups within the pathogen cluster: pathogenic group I and intermediate group II. These subgroups correlate with distinct virulence potentials (Thibeaux et al. 2018).

**Figure 1.**
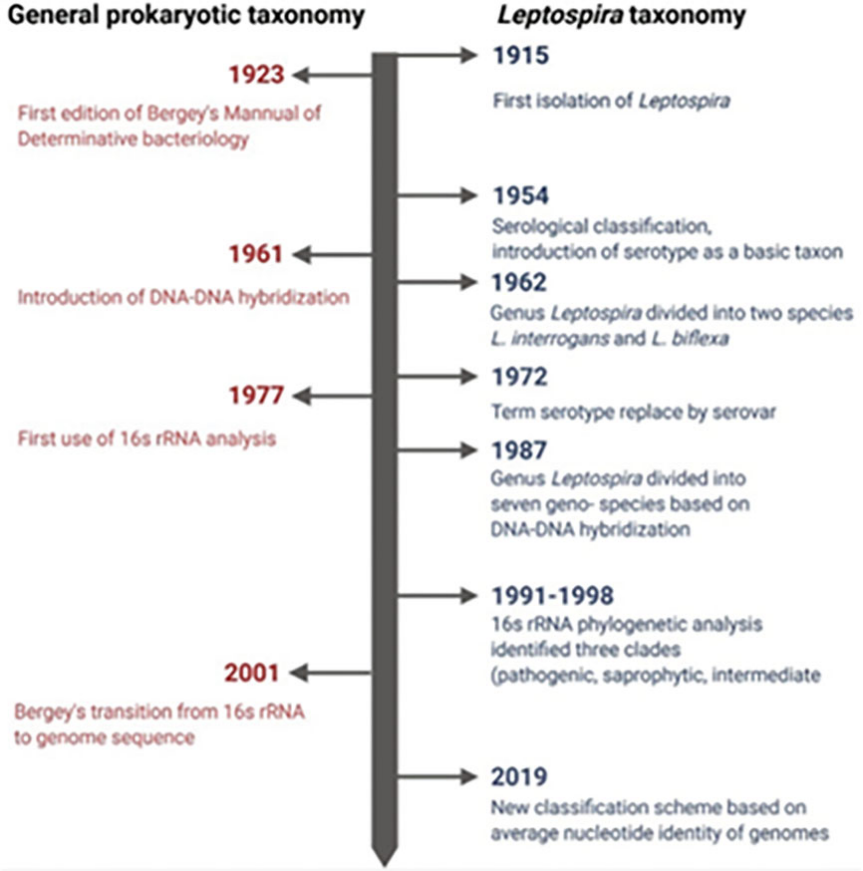
Key events in the evolution of systematics of prokaryotes and leptospires (Arent et al. 2022).

The key events in the development of *Leptospira* taxonomy, in relation to the broader classification of prokaryotic organisms, are outlined in Fig. 1 (Arent et al. 2022).

It should be noted that the diagnosis of leptospirosis requires specialized experience and can be complicated, potentially leading to misdiagnosis with severe consequences. Since the initial description of *Leptospira* by Stimson in 1907 (Stimson 1907), the genus *Leptospira* (L.) has traditionally been divided into two groups based on their pathogenicity: pathogens (*Leptospira interrogans* sensu lato) and saprophytes (*Leptospira* biflexa sensu lato) (Vincent et al. 2019).

Wolff and Broome pioneered the era of serological or antigenic classification by developing the microagglutination test (MAT), which allowed the differentiation of strains. This approach continued until 1954, when the cross-agglutinin absorption test (CAAT) was developed to separate numerous *Leptospira* serotypes (Wolff and Broome 1954).

A significant revolution in the classification of *Leptospira* occurred with the advent of methods based on the evolutionary divergence of nucleic acids—specifically DNA and RNA. This marked the beginning of the genetic era, enabled by the study of DNA-DNA hybridization, G + C content of DNA, and pulsed-field gel electrophoresis. These methods became crucial in determining the taxonomy and nomenclature of leptospires and established a reference standard for their phylogenetic classification (Wayne 1988). Woese and Fox significantly altered *Leptospira* taxonomy with their studies on the small subunit of ribosomal RNA (Woese and Fox 1977). Their work demonstrated that rRNA could provide an objective evolutionary framework, and the determination of the primary structure of 16S rRNA was more accessible than DNA hybridization.

The transition from phenotypic to phylogenetic classification was formalized in the Burge’s Manual of Systematic Bacteriology (Barco et al. 2020). Phylogenetic analysis of 16S rRNA sequences of *Leptospira* strains revealed three major clades: pathogenic, saprophytic, and intermediate (Cerqueira and Picardeau 2009, Ko et al. 2009).

The genomic era is relatively recent. Whole-genome sequencing (WGS) has revealed limitations in 16S rRNA sequence analysis, particularly its limited taxonomic resolution (Arent et al. 2022). The first WGS of the *Leptospira* genome became a powerful tool for bacterial strain classification and epidemiological typing (Guglielmini et al. 2019) and facilitated the development of new sequencing methods, such as next-generation sequencing (NGS). NGS is based on pairwise similarity measurements at the nucleotide (ANI) or protein (AAI) level (Konstantinidis and Tiedje 2005). This technology allowed for a more refined classification of the previously described clades. The pathogenic, intermediate, and saprophytic groups were further subdivided into sub-clades P1 and P2, and S1 and S2, respectively. P1 corresponds to the former pathogenic group, while P2 aligns with the former intermediate strains. Both S1 and S2 consist of the saprophytic group, but NGS revealed two genetically distinct clades, thus differentiating S1 and S2 (Vincent et al. 2019).

Currently, there remains a significant need for a systematic, standardized approach to describing bacterial genotype variations. Sequence variation in the genome serves as a key starting point for comparing different organisms, but efficient cataloguing of all types of genetic variation is essential for establishing comprehensive phenotype-genotype relationships. To meet this need, a new approach called core genome multilocus sequence typing (cgMLST) has been proposed. This method extends classical MLST to 545 core genes, representing the entire phylogenetic diversity of the genus (Guglielmini et al. 2019, Arent et al. 2022). One advantage of MLST is that it accurately reflects the genetic structure of *Leptospira* populations, is reproducible, and robustly supported by experimental data. It can even be used directly to identify leptospiral infections in clinical samples (Fouts et al. 2016).

In recent years, the *Leptospira* pan-genome project, supported by the National Institute of Allergy and Infectious Diseases (NIAID) Genome Sequencing Center, has provided the scientific community with hundreds of whole-genome sequences. This has enabled large-scale comparative genomic analyses, advancing our understanding of the genetic diversity and evolutionary dynamics of *Leptospira* spp. (Thibeaux et al. 2018).

Genome-wide analysis was able to identify a core set of genes shared by all isolates, along with a variable set of genes present in only a subset of isolates, and strain-specific or novel genes. This analysis is crucial for understanding the large-scale evolutionary mechanisms by which saprophytic bacteria acquire genes that confer infectivity and pathogenicity (Fouts et al. 2016).

The ability to distinguish between core and variable genes enhances our knowledge of how *Leptospira* strains adapt to new hosts, environments, and ecological niches. The data and insights generated by *Leptospira* WGS are poised to contribute to various critical areas of research, including the development of diagnostic tools, vaccines, and therapeutic strategies. These advances are particularly relevant within the framework of the One Health approach, which emphasizes the interconnectedness of human, animal, and environmental health. The continued exploration of the *Leptospira* genome will undoubtedly improve our understanding of leptospirosis and lead to more effective strategies to prevent and manage this important zoonotic disease.

### Environmental and animal hosts

Both pathogenic and saprophytic *Leptospira* strains have been isolated from environmental sources, demonstrating their ability to survive in moist soil and fresh water for extended periods. The survival of pathogenic leptospires in the environment is influenced by various factors, such as pH, temperature, and the presence of inhibitory compounds. This environmental persistence is critical for the transmission of leptospirosis, as the microorganisms can be reintroduced into the environment through the excreta of infected animals.

The ability of *Leptospira* to occupy diverse ecological niches is attributed to the large genome encoding multiple mechanisms that enable the organism to adapt and withstand stressful environmental conditions (Vincent et al. 2019). Okazaki and Ringen were the first in the field of soil microorganisms to investigate *Leptospira* survival and reported 6-month survival of virulent *Leptospira* in water-saturated soil (Okazaki and Ringen 1957).

Animals and humans can be classified as either supportive or accidental hosts. In supportive hosts, the infection is chronic and mostly localized in the renal tubules, where significant amounts of bacteria are shed into the environment via urine, where the pathogen can persist for long periods of time. Infected animals serve as a reservoir for the bacteria and contribute to its maintenance in nature. The most important supportive hosts for pathogenic leptospires are small mammals, such as rodents, which transmit the infection to domestic farm animals, dogs, and humans (Gomard et al. 2021).

A wide range of animals, including both domestic and wild species, serve as hosts for *Leptospira*, including rodents, small marsupials, pigs, horses, dogs, cattle, and even pinnipeds and bats. Birds, amphibians, reptiles, and cold-blooded animals (such as frogs, snakes, turtles, and potentially fish) can also harbor pathogenic leptospires (Lindtner-Knific et al. 2013, Hamond et al. 2015, Picardeau 2017).

Although humans can become infected from many sources, they are considered “dead-end hosts” in the transmission cycle, as the infection does not typically circulate among humans. However, human-to-human transmission is possible in rare cases, such as through organ transplantation, blood transfusion, or placental infection (Puliyath and Singh 2012, Song et al. 2016).

### Implications for diagnostic and control

The discovery of new strains of *Leptospira* underscores the need for ongoing improvements in diagnostic measures. It is essential to include new pathogenic serovars in diagnostic kits to enable faster, more accurate detection of the pathogen. Additionally, identifying and adapting treatment protocols for these evolving strains is vital in managing this severe disease.

A deeper understanding of *Leptospira* populations in the environment will be crucial in developing effective pathogen control strategies aimed at reducing infection risks. This research will also contribute to a more thorough understanding of bacterial ecology, adaptation, and evolutionary development, as well as their interactions with other microorganisms. Knowledge about the diversity of circulating *Leptospira* strains is essential for evaluating the effectiveness of current vaccines and for the development of new vaccine candidates.

Given the systemic nature of leptospirosis and the wide variety of target organs affected by the pathogen, the disease is extremely challenging from a medical perspective. Therefore, significant attention must be given to improve pathogen recognition and control measures. It is expected that the impact of global warming and climate change will contribute to the increased survival of *Leptospira* in the environment, as well as an increase in the survival and population density of its principal rodent hosts (Richardson et al 2025). This may lead to a higher incidence of infection in both humans and animals, making the issue of leptospirosis even more pressing in the context of public health.

From a public health standpoint, the discovery of new types of *Leptospira*—whether pathogenic, intermediate, or saprophytic—can have significant implications. Some studies indicate that genetic mechanisms like gene gain and genome reduction could facilitate the evolution of pathogenic strains from saprophytic ancestors (Thibeaux et al. 2018, Vincent et al. 2019), which could pose a direct threat to both human and animal populations, further emphasizing the importance of a “One Health” approach. Consequently, management and control strategies for this zoonotic pathogen remain challenging.

### Study objective

Given the complexity and evolving nature of leptospirosis, this study aims to:

- Provide an overview of the phylogenetic history of *Leptospira* spp.
- Summarize the most important diagnostic methods for detecting leptospirosis.
- Conduct a review and retrospective analysis of new strains discovered from 2000 to 2025, and include those that have significantly impacted the landscape of leptospirosis.

By addressing these points, this study seeks to contribute to the continued understanding and control of leptospirosis, a disease with far-reaching implications for both human and animal health.

## Materials and methods

This review was conducted to collect and analyze available data on the discovery and identification of novel *Leptospira* strains, with a particular focus on the geographic, ecological, and host-related origins of these strains.

### Literature search

A comprehensive literature search was conducted using the PubMed, Scopus, and ScienceDirect databases. In addition, data were obtained from two key repositories: the Leptospirosis Reference Center (Amsterdam, The Netherlands) and the German Collection of Microorganisms and Cell Cultures (Braunschweig, Germany).

The search strategy employed logical operators (“and” “or”) and combinations of the following keywords: *Leptospira* spp., *Leptospira* sp. nov., *Leptospira* and [country/region name], *Leptospira* and ecology, water, soil, environment, human, mammals, and rodents.

Articles were also manually reviewed to ensure inclusion of all relevant publications describing novel *Leptospira* strains or significant phylogenetic reclassifications published between 2000 and 2025.

### Study selection: inclusion and exclusion criteria

The selection process consisted of two phases. First, the titles and abstracts of search results were screened to remove duplicates and irrelevant studies. Second, full-text articles were reviewed based on predefined inclusion and exclusion criteria.

**Inclusion criteria:**

- Full-text articles in English.
- Published between 2000 and 2025.
- Studies describing the isolation, characterization, or genomic sequencing of new *Leptospira* strains (pathogenic, intermediate, or saprophytic).

**Exclusion criteria:**

- Conference abstracts, posters, or meeting notes.
- Non-peer-reviewed content.
- Reviews and theoretical modelling studies without primary strain data.

This systematic approach ensured that the review captured the most relevant discoveries of *Leptospira* spp. strain diversity over the last two decades, supporting a retrospective analysis of strain emergence and ecological distribution.

## Results and discussion

### Search results

Our database search yielded a total of 202 articles (60 from Scopus, 99 from PubMed, and 43 from ScienceDirect). After screening titles and abstracts, 121 articles were excluded. An additional 53 were excluded after full-text review. Four more relevant articles were identified manually. In total, 32 articles published between 2000 and 2025 were included in the analysis.

### Identified strains

In total, between 2000 and 2025, 89 novel *Leptospira* strains were selected, as shown in Supplement Tables S1–S3. To track the dynamics, all strains were divided by decade. Notably, 18 strains (20.2%) were reported in the first decade (Supplement Table S1), 53 strains (59.6%) were reported in the second decade (Supplement Table S2), and 18 strains (20.2%) between 2021 and 2025 (Supplement Table S3), highlighting a recent surge in discovery efforts. The peak falls on the 2011–2020 cluster, which, in our opinion, is due to the rapid development of the latest technologies for identifying and typing strains. Eighteen new strains have been selected as representatives of strain discoveries in the past five years. The number of discoveries will likely continue to increase, because WGS and the pan-genome project will help identify and characterize *Leptospira* strains that have not been studied until now (Lata et al. 2022). Of these: 59 strains (66.3%) belong to the pathogenic (P) clade, and 30 strains (33.7%) belong to the saprophytic (S) clade.

Many of these new isolates remain unassigned to specific serogroups or serovars, reflecting the growing reliance on genomic tools over classical serology for identification.

Modern whole-genome analysis has revealed that genetic mechanisms like gene gain and genome reduction could facilitate the evolution of pathogenic strains from saprophytic ancestors, expanding their ecological niche and increasing host range adaptability (Thibeaux et al. 2018, Vincent et al. 2019). This evolutionary plasticity reinforces the need to monitor environmental strains, as even non-pathogenic forms may present future public health risks.

### Geographic distribution of novel *Leptospira* strains

**Figure 2.**
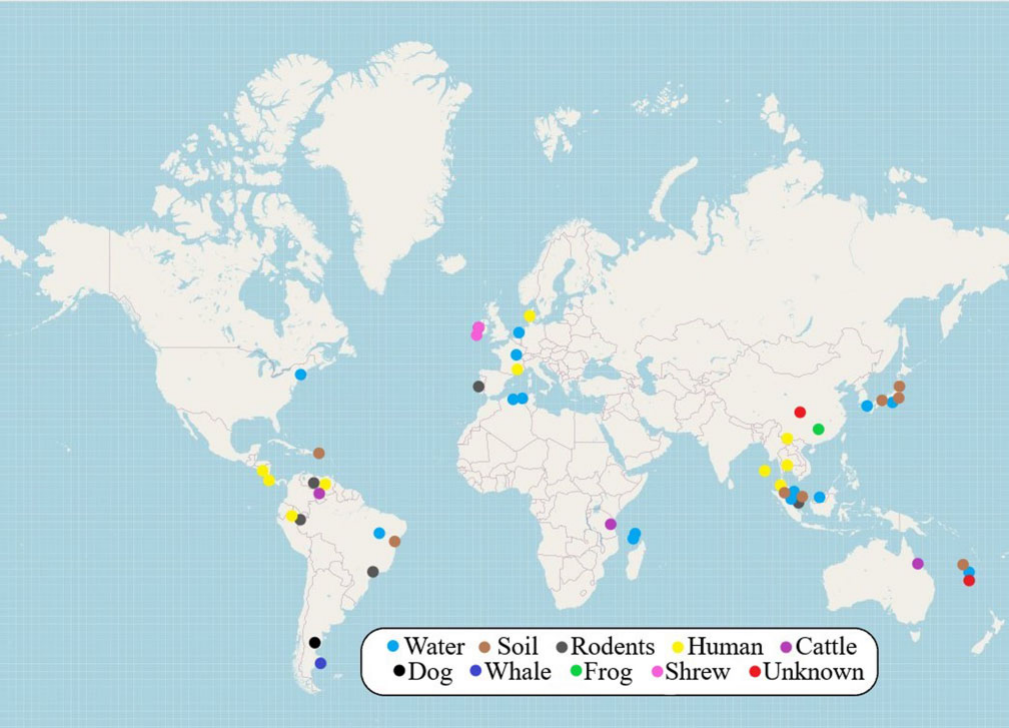
Geographical distribution of the *Leptospira* isolation sources. Map template obtained from OpenStreetMap.

New *Leptospira* strains included in the review were reported in 22 countries, across multiple regions (Fig. 2). Analyzing the geographical distribution of identified *Leptospira* strains, it was found that the largest number of strains was detected in European countries—32 (36%). In Asian countries, 27 strains (30.3%) and America—23 (25.8%). Countries of the African and Australian continents had the smallest number, 6 and 1, respectively. The highest numbers per country were found in New Caledonia—19 strains and Malaysia—16 strains. Other countries with moderate findings (5–6 strains) included Mayotte, Japan, and Algeria. In contrast, 1–3 strains were reported from countries such as Portugal, Costa Rica, France, Argentina, China, the USA, and others. This wide geographic distribution emphasizes the global relevance of leptospiral diversity and underscores the importance of regional surveillance.

### Sources and hosts of new *Leptospira* strains

The transmission cycle of leptospirosis involves an environmental reservoir, a susceptible host, and a route of transmission—primarily via water. Water not only facilitates leptospiral mobility but also plays a pivotal role in the persistence and spread of the pathogen in ecosystems (Thibeaux et al. 2018). Their survival in soil and water, especially in warm, humid climates, makes outbreaks particularly likely in tropical regions.

Rodents, as asymptomatic carriers, remain the primary reservoir hosts of pathogenic leptospires. Their widespread presence in human environments, particularly in low-income urban and rural settings, makes them central to transmission. In the pursuit of a One Health perspective, growing attention is being paid to the interplay between leptospires, animal reservoirs, and the environment.

In addition to rodents, *Leptospira* spp. have been isolated from domestic, farm, and wild animals, reinforcing their broad zoonotic potential (Assenga et al. 2015). The predominance of pathogenic (clade P) strains across all host sources highlights the ongoing risk to mammals and humans, while saprophytic (clade S) strains were isolated only from environmental sources.

#### Geographical source distribution

Geographical distribution and source of selected strains are shown in figure 2. The majority of *Leptospira* strains were isolated in proximity to water bodies, especially in tropical and subtropical climates. This spatial pattern correlates with higher precipitation, flooding events, and poor sanitation—factors that foster leptospiral survival and transmission (Levett 2001, Mwachui et al. 2015, Thibeaux et al. 2018, Bierque et al. 2020, Cunha et al. 2022).

However, sporadic discoveries in temperate zones (e.g. Netherlands, France, USA) suggest that climate change and rising temperatures may expand leptospiral habitats. This calls for enhanced surveillance and strain identification efforts in temperate ecosystems, which are likely to become increasingly vulnerable (Levett 2001, Pappas et al. 2008, Bandara et al. 2014, Allan et al. 2015, Costa et al. 2015, Soo et al. 2020, Cunha et al. 2022).

Outbreaks of the disease occur worldwide and more attention should be given to ecosystems in temperate climate zones as well. Given the worldwide abundance of leptospires and as climate in general is changing with increasing mean temperatures in temperate climate zones, it is worthwhile to intensify efforts and investigate *Leptospira* diversity and occurrence in these regions.

#### New strains isolated from water

As described previously, fresh water is an ideal environment and is the crucial element for survival and spread of leptospires, both saprophytic species that are regular inhabitants of this environment, and pathogenic ones (Davignon et al. 2023). Clade S is more widespread and most frequently isolated, making it difficult to distinguish it from clade P from water sources, as clade S strains usually replicate faster. Notably, Bierque et al. detected subclade P1 DNA in drinking water, raising concern over waterborne transmission even in treated sources (Bierque et al. 2020). Moreover, *Leptospira* spp. were also found in seawater, posing potential risks to marine ecosystems and fisheries, thereby possibly creating new ways for *Leptospira* to expand their host range even further (Ganoza et al. 2006, Bierque et al. 2020).

Freshwater environments remain a primary reservoir for both clade P and clade S leptospires, with 31 representative strains (34.8%) originating from water samples during the study period. Among them: 14 strains were pathogenic (clade P) and 17 strains were saprophytic (clade S). These isolates were reported from countries such as Malaysia (7), the USA (6), Algeria and Mayotte (5), New Caledonia (3), France, the Netherlands, Japan, India, and Brazil (1), respectively (Saito et al. 2013, Smythe et al. 2013, Masuzawa et al. 2019, Vincent et al. 2019, Casanovas-Massana et al. 2020, Korba et al. 2021, Sonam et al. 2024, Hamond et al. 2025).

These findings underscore that leptospires of varied virulence and ecology can occur in water bodies across diverse climates and geographies.

#### New *Leptospira* strains isolated from soil

The introduction of molecular techniques, particularly qPCR and genome sequencing, has allowed researchers to detect *Leptospira* in soil samples more accurately, particularly in swampy areas and post-flood environments (Davignon et al. 2023). Notably, clade P strains have been identified in urban soils in Brazil, Malaysia, and Taiwan (Fuh et al. 2011, Schneider et al. 2018, Casanovas-Massana et al. 2020, Philip et al. 2021, Sayanthi and Susanna 2024).

Over the past 25 years, 26 representative *Leptospira* strains have been isolated from soil (29.2% of total). These discoveries were made in countries including New Caledonia (15), Malaysia (5), Japan (3), Puerto Rico (2), and Brazil (1) (Slack et al. 2008, Thibeaux et al. 2018, Vincent et al. 2019, Masuzawa et al. 2019, Casanovas-Massana et al. 2020, Fernandes et al. 2022).

Approximately 60% of soil-derived strains belonged to the pathogenic clade P, emphasizing the epidemiological relevance of soil as a potential reservoir. In New Caledonia, 12 new *Leptospira* strains isolated from soil were recently identified by MALDI-ToF mass spectrometry and WGS analysis (Thibeaux et al. 2017). Another study by Thibeaux et al first demonstrated the success of PCR combined with molecular typing to identify strains and formally established that a pathogenic *Leptospira* strain remained viable for 9 weeks in a soil sample (Thibeaux et al. 2017, Thibeaux et al. 2018). These studies have evidenced changes in *Leptospira* diversity and revealed the possibility of presence of new unreported strains that were not or have not yet been found in mammals, which may indicate that soil too acts as an ecological reservoir for pathogenic *Leptospira*, creating protective conditions for their survival (Thibeaux et al. 2017).

Interestingly, several of these soil-derived strains have not yet been identified in mammals, suggesting the possibility of soil as an independent ecological reservoir for previously unrecognized pathogenic species. This line of research should be intensified, as it will be interesting to investigate relationship of pathogenic leptospires from soil and pathogenic leptospires from rodents, respectively.

#### New *Leptospira* strains in rodents

Urbanization and rapid expansion of urban areas have a deep impact on wildlife in general and on respective microbiomes in terms of adaptation to new conditions. Poor sanitation, mild winters, and increased accessibility and food waste create a breeding ground for rodents, which are the most important mammal species that maintain and spread leptospires worldwide (Mwachui et al. 2015, Philip et al. 2021). The *Rattus, Mus*, and *Apodemus* genera are most commonly involved. For example: *Leptospira interrogans* serogroup *Icterohaemorrhagiae* is typically hosted by *Rattus norvegicus*, and *L. borgpetersenii* serogroup *Ballum* is commonly found in *Mus musculus* (Levett 2001). Given the expansion behavior of some rodents and the capability of the *Leptospira* bacteria to adapt in these hosts fairly quickly, expansion of the host range can be expected. Leptospires demonstrate a broad host tropism, which enables them to adapt quickly to new species. For instance, recent findings include detection in Bank Voles (*Clethrionomys glareolus*) in Europe (Schmidt et al. 2021).

During the review period, eight new strains (9%) were isolated from rodents, all belonging to clade P. Noteworthy examples include: a Serovar Altodouro from *Mus musculus* in Portugal (Paiva-Cardoso et al. 2013); a Lyme-related strain isolated from *R. norvegicus* during an outbreak in Brazil (Moreno et al. 2018); a Serovar Varillal from *Rattus* spp. in the Peruvian Amazon (Matthias et al. 2008); and unnamed pathogenic strains from rodents detected near the housing of a human case in Venezuela (Puche et al. 2018) and Malaysia (Philip et al. 2021), respectively. In 2024, serovar Hardjo-bovis was identified in hamsters (*Cricetus cricetus)* for the first time in the USA (Putz et al. 2024).

These findings reaffirm the central role of rodents in maintaining and spreading pathogenic *Leptospira* and indicate the potential for further strain evolution and host adaptation.

#### New *Leptospira* strains in pets

Due to the large host tropism, it is well known that dogs can be reservoirs of pathogenic *Leptospira* with the capacity to infect humans. Infection of dogs often occurs through contact with water or soil contaminated with leptospires. The risk of infection for dog owners was confirmed by studies in the USA and Malaysia, respectively, where researchers analyzed clinically apparent dogs and found that those who had contact with wildlife, lived near bodies of water, bathed or drank the water were more likely to get infected (Rossetti et al. 2005, Schuller et al. 2015, Abdul Rahman, MS et al. 2021).

Although cats can be infected—most often via predation of infected rodents—clinical manifestations are rare. Still, some studies suggest they may shed leptospires in urine, posing a risk to humans (Schuller et al. 2015).

Between 2000 and 2025, a novel strain was isolated from an aborted canine fetus in Argentina. This strain (Baires) belongs to *Leptospira interrogans* (serogroup: Djasiman, serovar: Buenos Aires) (Rossetti et al. 2005). It is classified within clade P.

Since 2023 there is an ongoing outbreak of leptospirosis acquired from keeping of pet rats in Germany. Strain isolation is still ongoing (Stollberg et al. 2024).

#### New *Leptospira* strains in wild and farm animals

Wildlife hosts play a complex and often underappreciated role in the epidemiology of leptospirosis. Studies from Africa, Europe, and Latin America report high seroprevalence in mammals (31.4%), birds (27.8%), and reptiles (6.3%), underscoring their potential role as reservoirs of pathogenic leptospires (Allan et al. 2015, Assenga et al. 2015, Vieira et al. 2018). In France, Ayral et al. identified multiple serogroups in hedgehogs and martens; in Italy, *Leptospira* infection was detected in wild boars, foxes, hares, and ruminants; in Latin America, infections were confirmed in opossums (*Didelphis sp*.) and armadillos (*Euphractus sexcinctus, Dasypus novemcinctus*) (Ayral et al. 2016, Vieira et al. 2018).

Additionally, seropositivity and disease outbreaks have been reported in marine animals, including sea lions, fur seals, and manatees, where pathogenic strains have caused strandings and mortality (Grune Löffler et al. 2016).

Farm animals represent a well-known host group for *Leptospira spp*., particularly in tropical and subtropical regions. The pathogen is associated with reproductive failure, abortion, uveitis, and decreased productivity in cattle, pigs, horses, goats, and sheep (Levett 2001, Alinaitwe et al. 2024, 2025). Risk factors include exposure to wildlife, shared pastures, natural breeding, and access to contaminated water.

Based on literature analysis, between 2000 and 2025, 8 new strains (9%) have been identified: a strain Manara (*Leptospira brihuegai*) from a southern right whale (*Eubalaena australis*) in Argentina (Grune Löffler et al. 2016); three strains from a cattle—Sokoine in Tanzania (Mgode et al. 2006), Topaz in Australia (Corney et al. 2008) and IVIC-Bov1 in Venezuela (Puche et al. 2018); two strains in Ireland—Room22 (*L. alstonii*) from a white-toothed shrew (*Crocidura russula*) (Nally et al. 2016) and GWTS#1^T^ from a house shrew (*Crocidura russula*) (Vincent et al. 2019); a serovar Sichuan from a frog in China (Smythe et al. 2013); a strain RedPanda1 from a red panda (*Ailurus fulgens*) in the USA (LeCount et al. 2023).

Interestingly, all the identified strains belong to the intermediate clade P, suggesting a possible role for farm animals in maintaining these environmentally adapted strains with potential pathogenicity.

#### New Leptospira strains in humans

Humans are considered accidental or incidental hosts in the leptospiral transmission cycle, typically becoming infected through direct or indirect contact with contaminated water, soil, or infected animals. Although human-to-human transmission is extremely rare, it has been reported, for example, via blood transfusion (Ribeiro et al. 1997, Tulsiani et al. 2010). Most cases of leptospirosis in humans are associated with occupational or recreational exposure and severe weather events such as flooding. High-risk occupation groups include: veterinarians, abattoir workers, sewage treatment workers, farmers, and agricultural laborers, especially in tropical regions (e.g. rice and banana plantation workers), and recreational exposure: individuals participating in water sports (e.g. kayaking, rafting, triathlons) or outdoor activities near natural water sources (e.g. fishing, caving, hunting) (Haake and Levett 2015).

Between 2000 and 2025, 12 representative pathogenic *Leptospira* strains (13.5%) were identified in human patients (Table 1).

**Table 1.** Representative pathogenic *Leptospira* strains from human cases.

Strain	Species	Country	Reference
Arenal	*L. santarosai*	Costa Rica	Valverde et al. (2008)
Broomii	*L. broomii*	Denmark, France	Levett et al. (2006)
Heyan	*L. weilii*	China	Xu et al. (2017)
Hurstbridge type HB6	*L. fainei*	Denmark	Petersen et al. (2001)
Khorat	*L. wolffii*	Thailand	Slack et al. (2008)
Langkawi (Lai type)	*L. interrogans*	Malaysia	Wagenaar et al. (2004)
Portblairi	*L. interrogans*	India	Vijayacharit et al. (2004)
Varillal	*L. licerasiae*	Peru	Matthias et al. (2008)
CLM-U50^T^	*L. venezuelensis*	Venezuela	Puche et al. (2018)
200901116^T^	*L. mayottensis*	Mayotte	Bourhy et al. (2014)
Corredores	*L. santarosai*	Costa Rica	Valverde et al. (2013)
Costa Rica	*L. santarosai*	Costa Rica	Valverde et al. (2013)

All of these isolates were identified as members of the pathogenic clade P, underscoring the public health significance of emerging human-pathogenic *Leptospira* strains.

It should be noted, that over the past 5 years, there have been no cases of *Leptospira* identification in people who had leptospirosis (Supplement Table S3).

#### Other new Leptospira strains with unknown sources

Two newly described strains were identified in recent decades for which no specific source of isolation was documented: a serovar Hualin, *Leptospira terpstrae* (serogroup: *Icterohaemorrhagiae*), isolated in China, described by Smythe et al. (2013), and a strain PZF5-3T, *Leptospira montravelensis*, discovered in New Caledonia by Vincent et al. (2019).

Both strains were classified within the saprophytic clade S, suggesting environmental origin, although definitive ecological sources remain unknown.

## Conclusion

Leptospirosis has gained increased attention in recent decades due to its growing impact on global public health. Rising morbidity and mortality rates underscore its significance as a re-emerging zoonotic disease (Beauté et al. 2024). The pathogen *Leptospira* exhibits extensive species and serovar diversity, multiple routes of transmission, and a broad range of reservoir hosts, which collectively complicate its surveillance, diagnosis, and control.

Besides known disease drivers like rainy seasons and flooding anthropogenic factors—such as rapid urban expansion, inadequate sanitation, mismanagement of food waste, and low public awareness—continue to contribute to the increasing frequency of outbreaks, particularly in urban and peri-urban areas. Furthermore, climate change, through rising temperatures and milder winters, is expanding the ecological range of *Leptospira*, making temperate regions increasingly susceptible to transmission.

Since 2010, the discovery of numerous novel *Leptospira* strains has significantly advanced our understanding of the pathogen’s ecology and evolution. These new isolates, identified in diverse environmental matrices—soil, water, animal hosts, and humans—highlight the organism’s adaptive potential and reinforce the need for continued One Health-based surveillance. In parallel, molecular techniques such as WGS and NGS have revolutionized *Leptospira* taxonomy, enabling the identification of previously uncharacterized or misclassified species.

This review has consolidated current knowledge on newly described *Leptospira* strains and mapped their global and ecological distribution. The data demonstrate that both pathogenic (clade P) and saprophytic (clade S) leptospires are widely present across ecosystems, with water and soil playing a critical role in their persistence and transmission. Importantly, the detection of new strains in hosts ranging from rodents and domestic animals to marine mammals and humans underlines the importance of a multisectoral approach to disease monitoring and control.

In conclusion, the ongoing discovery of novel *Leptospira* strains calls for sustained epidemiological surveillance, especially in regions vulnerable to environmental and climatic shifts. Understanding the ecological interactions, host adaptation, and environmental survival strategies of *Leptospira* is essential for improving diagnostic tools, risk assessment, and the development of targeted prevention and control strategies. Future research efforts must continue to integrate human, animal, and environmental health perspectives to effectively address the challenges posed by this globally significant pathogen.

## Supplementary Material

fuaf054_Supplemental_Files
