# Supplementary material for: Look and you will find—a literature review of new strains of Leptospira spp., 2000–2025
Source: FEMS Microbiol Rev. 2025 Nov 6;49:fuaf054. doi: 10.1093/femsre/fuaf054 (PMC12629226; doi:10.1093/femsre/fuaf054)
Supplement: fuaf054_Supplemental_Files [file fuaf054_supplemental_files.zip › Supplement Table 1.docx]

Table 1. **List of new leptospira strains isolated from 2000–2010 included in the study**

| **Leptospira taxonomy** | | | **Source** | **Country (region)** | **Year of isolation** | **References** |
| --- | --- | --- | --- | --- | --- | --- |
| **Serovar** | **Strain** | **Serogroup / Species** |  |  |  |  |
| Altodouro | RIM 139 | *Pomona / L. kirschneri* | house mouse  (*Mus musculus*) | Northern Portugal  (Tras-os-Montes e Alto Douro region) | 2009 | Paiva-Cardoso  et al., 2013 |
| Arenal | MAVJ 401 | *Javanica / L. santarosai* | human  (biologist at fish farm) | Costa Rica  (Alajuela province) | 2007 | Valverde  et al., 2008 |
| Broomii | 5399^T^ | - / *L. broomii* | human  (blood, urine and cerebrospinal fluid) | Denmark,  France (Marseilles) | 2000, 2001 | Levett et al., 2006 |
| Buenos Aires | Baires | *Djasiman / L. interrogans* | dog  (aborted fetus) | Argentina | 2005 | Rossetti  et al., 2005 |
| Heyan | L231 | *Manhao / L. weilii* | human | China (Yunnan) | 2004 | Xu et al., 2017 |
| Hurstbridge  type HB6 | 5399 | *Hurstbridge / L. fainei* | human  (cleaning aircraft at Danish airport) | Denmark | 2001 | Petersen  et al., 2001 |
| Khorat | Khorat-H2^T^ | - / *L. wolffii* | human  (urine) | Thailand  (province of Nakornra-chasima (Khorat) | 2008 | Slack et al.,  2008 |
| Lai type Langkawi | Langkawi | *Icterohaemorrhagiae / L. interrogans* | human  (after returning  from vacation) | Malaysia  (Langkawi Island) | 2004 | Wagenaar  et al., 2004 |
| Malaysia | Bejo-Iso9^T^ | *Tarassovi / L. kmetyi* | soil | Malaysia (Johor) | 2009 | Slack et al., 2009 |
| Manara | Manara | - / *L. brihuegai* | Southern right whale (*Eubalaena australis*) | Argentina  (Playa Manara,  Península Valdés) | 2010 | Grune Loffler et al., 2016 |
| Portblairi | DS2 | *Sehgali / L. interrogans* | human (blood) | India (North Andaman) | 2004 | Vijayacharit et al., 2004 |
| Sokoine | RM1 | *Icterohaemorrhagiae / L. kirschneri* | cattle  (cow from dairy farm) | Tanzania  (slaughterhouse in Morogoro) | 2005 | Mgode et al., 2006 |
| Topaz | 94-79970/3 | *Tarassovi / L. weilii* | cattle  (heifer's urine) | Australia  (Northern Queensland) | 2008 | Corney  et al., 2008 |
| Varillal | VAR 010^T^ | *Iquitos / L. licerasiae* | human (blood),  rat  (*R. norvegicus*; *R. rattus*) | Peru  (Peruvian Amazon,  Iquitos region) | 2005 | Matthias  et al., 2008 |
| unknown | CLM-U50^T^ | unknown / *L. venezuelensis* | human  (urine) | Venezuela | 2010 | Puche et al., 2018 |
| unknown | CLM-R50 | unknown / *L. venezuelensis* | rat  (*R. norvegicus*) | Venezuela | 2010 | Puche et al., 2018 |
| unknown | IVIC-Bov1 | unknown / *L. venezuelensis* | cow  (*Bos taurus*) | Venezuela | 2010 | Puche et al., 2018 |
| undesignated* | 200901116^T^ | *Mini*/ *L. mayottensis* | human  (blood) | Mayotte  (French island in the Indian Ocean) | 2009 | Bourhy  et al., 2014 |
|  |  |  |  |  |  |  |

*Undesignated indicates that a serogroup or/and serovar have not been assigned yet and association to species remains to be validated by proposed ILS criteria (Nally, et al. 2023).

Nally J, Galloway R, Picardeau M *et al.* Position Statement – Speciation of Leptospiral Isolates and Minimum Criteria for Species Definition. In: Society IL (ed.): 1 Edition: International Leptospirosis Society, 2023.
