# Supplementary material for: Look and you will find—a literature review of new strains of Leptospira spp., 2000–2025
Source: FEMS Microbiol Rev. 2025 Nov 6;49:fuaf054. doi: 10.1093/femsre/fuaf054 (PMC12629226; doi:10.1093/femsre/fuaf054)
Supplement: fuaf054_Supplemental_Files [file fuaf054_supplemental_files.zip › Supplement Table 2.docx]

**Table 2. List of new leptospira strains isolated from 2011–2020 included in the study**

| **Leptospira taxonomy** | | | **Source** | **Country (region)** | **Year of isolation** | **References** |
| --- | --- | --- | --- | --- | --- | --- |
| **Serovar** | **Strain** | **Serogroup / Species** |  |  |  |  |
| Corredores | JICH 05 | *Tarassovi / L. santarosai* | human  (after swimming in Corredores River) | Costa Rica  (Corredores canton) | 2013 | Valverde  et al, 2013 |
| Costa Rica | INCIENSA 04 | *Pyrogenes / L. santarosai* | human  (after swimming in  La Bonita Brook River) | Costa Rica  (Corredores canton) | 2013 | Valverde  et al., 2013 |
| Holland | WazHolland^T^ | *Holland / L. vanthielii* | water | Netherlands | 2013 | Smythe  et al., 2013 |
| Hualin | LT 11-33^T^ | *Icterohaemorrhagiae / L. terpstrae* | unknown | China | 2013 | Smythe  et al., 2013 |
| Lyme | M34/99 | *Lyme / L. inadai* | urban brown rat  (*R. norvegicus*) | Brazil  (São Paulo) | 2018 | Moreno  et al, 2018 |
| Room22 | GWTS#1 | undesignated / *L. alstonii* | Greater white  toothed shrew  (*Crocidura russula*) | Ireland | 2016 | Nally et al.,  2016 |
| Saopaulo | Sao Paulo^T^ | *Semaranga / L. yanagawae* | water | Brazil | 2013 | Smythe  et al., 2013 |
| Sichuan | 79601^T^ | undesignated / *L. alstonii* | frog | China | 2013 | Smythe  et al., 2013 |
| unknown | F1^T^ | unknown / *L. yasudae* | topsoil  (urban slum community of Pau da Lima) | Brazil (Salvador) | 2015 | Casanovas-Massana et al., 2020 |
| unknown | Yale^T^ | unknown/ *L. stimsonii* | water  (Mills River) | USA  (New Haven, Connecticut) | 2016 | Casanovas-Massana et al., 2020 |
| undesignated* | ATI7-C-A5^T^ | undesignated* / *L. ellisii* | soil | New Caledonia  (South Province) | 2017 | Thibeaux  et al., 2018 |
| undesignated* | FH4-C-A1^T^ | undesignated* / *L. barantonii* | soil | New Caledonia  (North Province) | 2017 | Thibeaux  et al., 2018 |
| undesignated* | FH2-B-D1^T^ | undesignated* / *L. adleri* | soil | New Caledonia  (North Province) | 2017 | Thibeaux  et al., 2018 |
| undesignated* | FH1-B-B1 | undesignated* / *L. perolatii* | soil | New Caledonia  (North Province) | 2017 | Thibeaux  et al., 2018 |
| undesignated* | ES4-C-A1^T^ | undesignated* / *L. neocaledonica* | soil | New Caledonia  (North Province) | 2017 | Thibeaux  et al., 2018 |
| undesignated* | FH4-C-A2^T^ | undesignated* / *L. saintgironsiae* | soil | New Caledonia  (North Province) | 2017 | Thibeaux  et al., 2018 |
| undesignated* | ATI7-C-A4^T^ | undesignated* / *L. haakeii* | soil | New Caledonia  (South Province) | 2017 | Thibeaux  et al., 2018 |
| undesignated* | MCA2-B-A3^T^ | undesignated* / *L. hartskeerlii* | soil | New Caledonia  (North Province) | 2017 | Thibeaux  et al., 2018 |
| undesignated* | FH2-B-A1^T^ | undesignated* /*L. harrisiae* | soil | New Caledonia  (North Province) | 2017 | Thibeaux  et al., 2018 |
| undesignated* | MCA2-B-A1^T^ | undesignated* / *L. levettii* | soil | New Caledonia  (North Province) | 2017 | Thibeaux  et al., 2018 |
| undesignated* | JW2-C-A2^T^ | undesignated* /*L. brenneri* | soil | New Caledonia  (North Province) | 2017 | Thibeaux  et al., 2018 |
| undesignated* | ATI2-C-A1^T^ | undesignated* / *L. macculloughii* | soil | New Caledonia  (North Province) | 2017 | Thibeaux  et al., 2018 |
| undesignated* | Eri-1^T^ | undesignated* /*L. idonii* | water | Japan  (Kyushu University, Fukuoka) | 2013 | Saito et al., 2013 |
| undesignated* | M12A^T^ | undesignated* /*L. dzianensis* | water  (Dziani Lake) | Mayotte | 2019 | Vincent  et al., 2019 |
| undesignated* | GWTS#1^T^ | undesignated* / *L. tipperaryensis* | house shrew  (*Crocidura russula*) | Ireland  (Tipperary county) | 2019 | Vincent  et al., 2019 |
| undesignated* | KG8-B22^T^ | undesignated* / *L. gomenensis* | soil | New Caledonia  (Kaala-Gomen village) | 2019 | Vincent  et al., 2019 |
| undesignated* | SSW20^T^ | undesignated* / *L. putramalaysiae* (later heterotypic synonym of *L. stimsonii*) | water | Malaysia  (Sungai Congkak) | 2019 | Vincent  et al., 2019 |
| undesignated* | PZF11-2^T^ | undesignated* / *L. andrefontaineae* | water | New Caledonia (Nouméa) | 2019 | Vincent  et al., 2019 |
| undesignated* | M11A^T^ | undesignated* / *L. dzoumogneensis* | water | Mayotte  (Dzoumogné village) | 2019 | Vincent  et al., 2019 |
| undesignated* | TK1-4^T^ | undesignated* / *L. koniamboensis* | water | New Caledonia  (Koné, North Province) | 2019 | Vincent  et al., 2019 |
| undesignated* | LIMR175^T^ | undesignated* / *L. sarikeiensis* | water | Malaysia  (Sarawak, Sarikei district) | 2019 | Vincent  et al., 2019 |
| undesignated* | SCS5^T^ | undesignated* /*L. fluminis* | soil | Malaysia  (Sungai Congkak) | 2019 | Vincent  et al., 2019 |
| undesignated* | SSW15^T^ | undesignated* / *L. fletcheri* | water | Malaysia  (Sungai Congkak) | 2019 | Vincent  et al., 2019 |
| undesignated* | SSS9^T^ | undesignated* /*L. semungkisensis* | soil | Malaysia  (Sungai Congkak) | 2019 | Vincent  et al., 2019 |
| undesignated* | SSW18^T^ | undesignated* / *L. langatensis* | water | Malaysia  (Langat district,  Sungai Congkak) | 2019 | Vincent  et al., 2019 |
| undesignated* | SSW17^T^ | undesignated* / *L. selangorensis* | water | Malaysia  (Sungai Congkak) | 2019 | Vincent  et al., 2019 |
| undesignated* | 201702476^T^ | undesignated* / *L. ognonensis* | water | France  (Bourgogne-Franche-Comté) | 2019 | Vincent  et al., 2019 |
| undesignated* | 201400974^T^ | undesignated* / *L. ilyithenensis* | water | Algeria  (Ilyithen village) | 2019 | Vincent  et al., 2019 |
| undesignated* | M10A^T^ | undesignated* / *L. bandrabouensis* | water | Mayotte  (Bandraboua commune) | 2019 | Vincent  et al., 2019 |
| undesignated* | PZF14-4^T^ | undesignated* / *L. noumeaensis* | water | New Caledonia  (Nouméa city,  South Province) | 2019 | Vincent  et al., 2019 |
| undesignated* | L5S1^T^ | undesignated* /*L. jelokensis* | soil | Malaysia  (Sungai Jelok) | 2019 | Vincent  et al., 2019 |
| undesignated* | PZF7-6^T^ | undesignated* / *L. bourretii* | soil | New Caledonia  (Nouméacity,  South Province) | 2019 | Vincent  et al., 2019 |
| undesignated* | TK5-11^T^ | undesignated* /*L. kanakyensis* | soil | New Caledonia  (Koné, North Province) | 2019 | Vincent  et al., 2019 |
| undesignated* | LIMR131^T^ | undesignated* / *L. kemamanensis* | water | Malaysia  (Terengganu,  Kemaman district) | 2019 | Vincent  et al., 2019 |
| undesignated* | M2A^T^ | undesignated* /*L. mtsangambouensis* | water | Mayotte  (Mtsangamboua village) | 2019 | Vincent  et al., 2019 |
| undesignated* | M1A^T^ | undesignated* /*L. bouyouniensis* | water | Mayotte  (Bouyouni village) | 2019 | Vincent  et al., 2019 |
| undesignated* | SCS9^T^ | undesignated* /*L. congkakensis* | soil  (a recreational forest) | Malaysia  (Sungai Congkak) | 2019 | Vincent  et al., 2019 |
| undesignated* | HP2^T^ | undesignated* /*L. perdikensis* | water  (a waterfall Perdik) | Malaysia  (Hulu Perdik) | 2019 | Vincent  et al., 2019 |
| undesignated* | PZF5-3^T^ | undesignated* / *L. montravelensis* | unknown | New Caledonia  (Montravel district, Nouméa) | 2019 | Vincent  et al., 2019 |
| undesignated* | E8^T^ | undesignated* /*L. johnsonii* | soil  (a rice field) | Japan  (Kamisu City, Ibaraki Prefecture) | 2018 | Masuzawa et al., 2019 |
| undesignated* | E30^T^ | undesignated* /*L. kobayashii* | soil | Japan (Gifu) | 2018 | Masuzawa et al., 2019 |
| undesignated* | YH101^T^ | undesignated* / *L. ryugenii* | surface water  (a botanical garden) | Japan  (Shizuoka City,  Shizuoka Prefecture) | 2018 | Masuzawa et al., 2019 |
| undesignated* | E18^T^ | undesignated* /*L. ellinghausenii* | soil  (in the park) | Japan  (Sukagawa City,  Fukushima Prefecture) | 2018 | Masuzawa et al., 2019 |

*Undesignated indicates that a serogroup or/and serovar have not been assigned yet and association to species remains to be validated by proposed ILS criteria (Nally, et al. 2023).

Nally J, Galloway R, Picardeau M *et al.* Position Statement – Speciation of Leptospiral Isolates and Minimum Criteria for Species Definition. In: Society IL (ed.): 1 Edition: International Leptospirosis Society, 2023.
