# Supplementary material for: Look and you will find—a literature review of new strains of Leptospira spp., 2000–2025
Source: FEMS Microbiol Rev. 2025 Nov 6;49:fuaf054. doi: 10.1093/femsre/fuaf054 (PMC12629226; doi:10.1093/femsre/fuaf054)
Supplement: fuaf054_Supplemental_Files [file fuaf054_supplemental_files.zip › Supplement Table 3.docx]

**Table 3.** **List of new leptospira strains isolated from 2021–2025 included in the study**

| **Leptospira taxonomy** | | | **Source** | **Country (region)** | **Year of isolation** | **References** |
| --- | --- | --- | --- | --- | --- | --- |
| **Serovar** | **Strain** | **Serogroup / Species** |  |  |  |  |
| unknown | LGVF01 | *- / L. sanjuanensis* | soil | Puerto Rico (San Juan) | 2022 | Fernandes  et al., 2022 |
| unknown | LGVF02^T^ | *- / L. sanjuanensis* | soil | Puerto Rico (San Juan) | 2022 | Fernandes  et al., 2022 |
| unknown | HP 364 | unknown / *L. borgpetersenii* | rodents  (trapped in human leptospirosis outbreak premises) | Malaysia | 2021 | Philip et al., 2021 |
| unknown | HP 358 | unknown / *L. interrogans* | rodent  *Sundamys muelleri*  (trapped in human leptospirosis suspected area, in forest environment) | Malaysia  (Hulu Perdik, Selangor) | 2021 | Philip et al., 2021 |
| unknown | SC 295 | unknown / *L. weilii* | rodents  (trapped in human leptospirosis outbreak premises) | Malaysia | 2021 | Philip et al., 2021 |
| undesignated* | 201903070^T^ | undesignated* / *L. ainlahdjerensis* | water  (near cattle farm) | Algeria  (Aïn Lahdjer region,  province of Sétif) | 2021 | Korba et al., 2021 |
| undesignated* | 201903071^T^ | undesignated* / *L. ainazelensis* | water  (near cattle farm) | Algeria  (Aïn Azel region,  province of Sétif) | 2021 | Korba et al., 2021 |
| undesignated* | 201903074^T^ | undesignated* / *L. abararensis* | water | Algeria  (Chréa region,  province of Blida) | 2021 | Korba et al., 2021 |
| undesignated* | 201903075^T^ | undesignated* / *L. chreensis* | water | Algeria  (Chréa region,  province of Blida) | 2021 | Korba et al., 2021 |
| unknown | WS4.C2^T^ | unknown / *L. mgodei* | water | USA (Iowa) | 2025 | Hamond et al., 2025 |
| unknown | WS39.C2^T^ | unknown / *L. iowaensis* | water | USA (Iowa) | 2025 | Hamond et al., 2025 |
| unknown | WS58.C2^T^ | unknown / *L. cinconiae* | water | USA (Iowa) | 2025 | Hamond et al., 2025 |
| unknown | WS60.C2^T^ | unknown / *L. milleri* | water | USA (Iowa) | 2025 | Hamond et al., 2025 |
| unknown | WS92.C1^T^ | unknown / *L. gorisiae* | water | USA (Iowa) | 2025 | Hamond et al., 2025 |
| unknown | KeTo | unknown / *L. interrogans* | sewage water  (a wildlife enclosure) | India (Mangalore) | 2024 | Sonam et al., 2024 |
| Hardjo-bovis | JB197 | *L. borgpetersenii* | hamster  (*Cricetus cricetus*) | USA | 2024 | Putz et al., 2024 |
| Hardjo-bovis | HB203 | *L. borgpetersenii* | hamster  (*Cricetus cricetus*) | USA | 2024 | Putz et al., 2024 |
| Grippotyphosa | RedPanda1 | *L. kirschneri* | red panda  (*Ailurus fulgens*) | USA | 2023 | LeCount et al., 2023 |
|  |  |  |  |  |  |  |

*Undesignated indicates that a serogroup or/and serovar have not been assigned yet and association to species remains to be validated by proposed ILS criteria (Nally, et al. 2023).

Nally J, Galloway R, Picardeau M *et al.* Position Statement – Speciation of Leptospiral Isolates and Minimum Criteria for Species Definition. In: Society IL (ed.): 1 Edition: International Leptospirosis Society, 2023.
